# Supplementary material for: The microbiome biomarkers of pregnant women’s vaginal area predict preterm prelabor rupture in Western China
Source: Front Cell Infect Microbiol. 2024 Oct 31;14:1471027. doi: 10.3389/fcimb.2024.1471027 (PMC11560878; doi:10.3389/fcimb.2024.1471027)
Supplement: Supplementary file 1 [file DataSheet1.zip › compare_1/Community/Pieplot/species/sample/C9.species.pieplot.pdf]

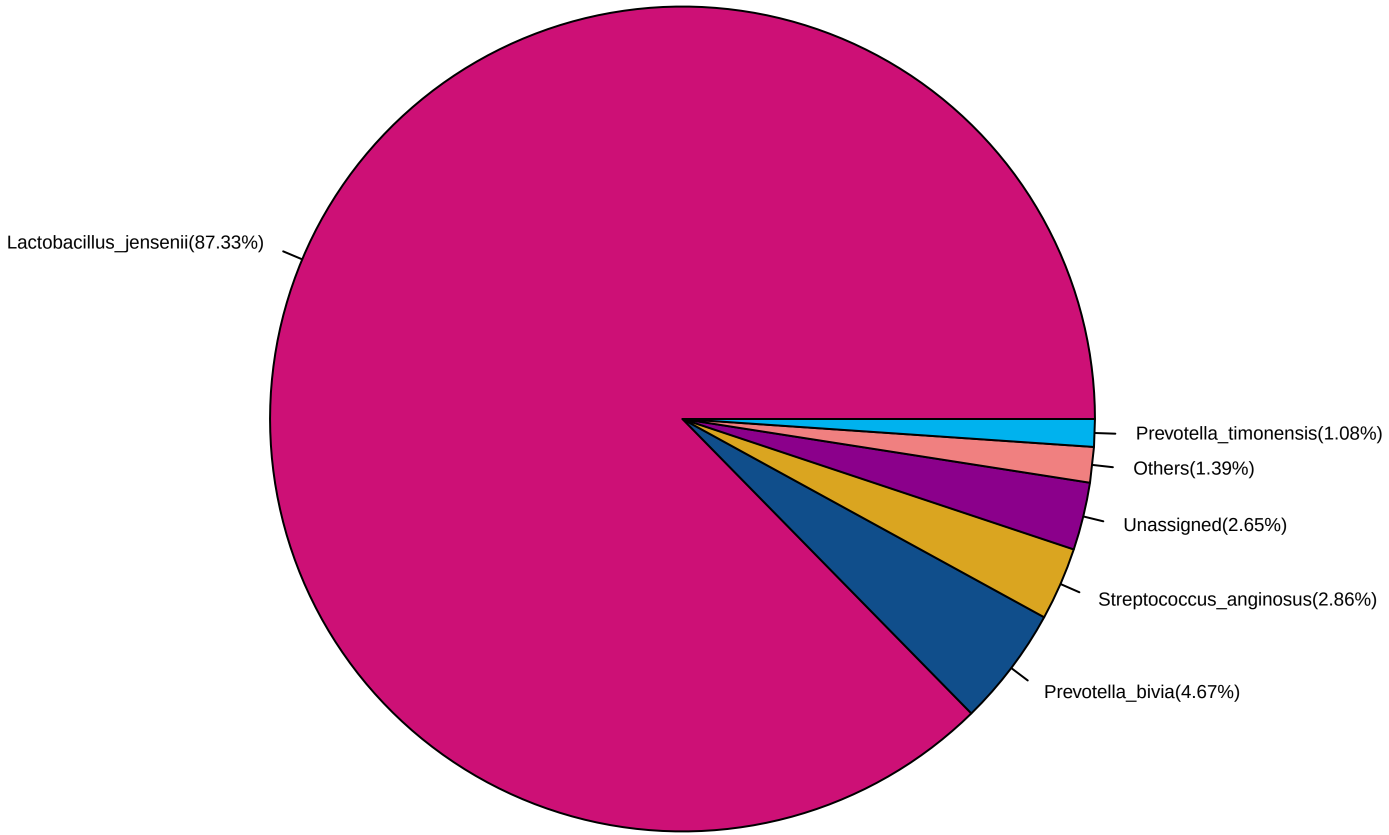
Lactobacillus_jensenii(87.33%)
Prevotella_timonensis(1.08%)
Others(1.39%)
Unassigned(2.65%)
Streptococcus_anginosus(2.86%)
Prevotella_bivia(4.67%)
